# Spinal Cord Metabolic Signatures in Models of Fast- and Slow-Progressing SOD1^G93A^ Amyotrophic Lateral Sclerosis

**DOI:** 10.3389/fnins.2019.01276

**Published:** 2019-12-10

**Authors:** Gabriel N. Valbuena, Lavinia Cantoni, Massimo Tortarolo, Caterina Bendotti, Hector C. Keun

**Affiliations:** ^1^Department of Surgery and Cancer, Imperial College London, London, United Kingdom; ^2^Department of Molecular Biochemistry and Pharmacology, Istituto di Ricerche Farmacologiche Mario Negri IRCCS, Milan, Italy; ^3^Department of Neuroscience, Istituto di Ricerche Farmacologiche Mario Negri IRCCS, Milan, Italy

**Keywords:** amyotrophic lateral sclerosis (ALS), SOD1^G93A^ ALS mouse model, metabolism, metabolomics, spinal cord, oxidative stress, TCA cycle, energy metabolism

## Abstract

The rate of disease progression in amyotrophic lateral sclerosis (ALS) is highly variable, even between patients with the same genetic mutations. Metabolic alterations may affect disease course variability in ALS patients, but challenges in identifying the preclinical and early phases of the disease limit our understanding of molecular mechanisms underlying differences in the rate of disease progression. We examined effects of SOD1^G93A^ on thoracic and lumbar spinal cord metabolites in two mouse ALS models with different rates of disease progression: the transgenic SOD1^G93A^-C57BL/6JOlaHsd (C57-G93A, slow progression) and transgenic SOD1^G93A^-129SvHsd (129S-G93A, fast progression) strains. Samples from three timepoints (presymptomatic, disease onset, and late stage disease) were analyzed using Gas Chromatography-Mass Spectrometry metabolomics. Tissue metabolome differences in the lumbar spinal cord were driven primarily by mouse genetic background, although larger responses were observed in metabolic trajectories after the onset of symptoms. The significantly affected lumbar spinal cord metabolites were involved in energy and lipid metabolism. In the thoracic spinal cord, metabolic differences related to genetic background, background-SOD1 genotype interactions, and longitudinal SOD1^G93A^ effects. The largest responses in thoracic spinal cord metabolic trajectories related to SOD1^G93A^ effects before onset of visible symptoms. More metabolites were significantly affected in the thoracic segment, which were involved in energy homeostasis, neurotransmitter synthesis and utilization, and the oxidative stress response. We find evidence that initial metabolic alterations in SOD1^G93A^ mice confer disadvantages for maintaining neuronal viability under ALS-related stressors, with slow-progressing C57-G93A mice potentially having more favorable spinal cord bioenergetic profiles than 129S-G93A. These genetic background-associated metabolic differences together with the different early metabolic responses underscore the need to better characterize the impact of germline genetic variation on cellular responses to ALS gene mutations both before and after the onset of symptoms in order to understand their impact on disease development.

## Introduction

Amyotrophic lateral sclerosis (ALS) is a rare, fatal neurodegenerative disease characterized by a progressive loss of motor neurons in the spinal cord, brainstem and cerebral cortex. The majority of ALS cases are classified as sporadic (sALS, about 90%) while the remaining 10% are familial (fALS), resulting from inherited mutations in more than 20 genes (Chia et al., 2018). However, we are currently unable to distinguish between the two based on clinical presentation and using existing technologies.

The clinical presentation and course of ALS is markedly heterogeneous. Most ALS patients are only highlighted as probable cases after some aspect of motor function has already been compromised, and their condition would have already deteriorated significantly by the time these diagnoses are confirmed. The rate of disease progression is also highly variable among different patients (Chió et al., 2011; Pupillo et al., 2014; Fournier and Glass, 2015) even between those with the same genetic familial form (Regal et al., 2006). A combination of genetic and environmental or exogenous modifying factors are believed to underlie the variability in disease progression. While there are a number of accurate machine learning models of ALS progression proposed that include rates of decline in functional measures (Kuffner et al., 2015; Westeneng et al., 2018), there are currently no standardized biomarkers or diagnostic protocols at diagnosis to predict how rapidly patients are likely to deteriorate. Together, the inability to identify individuals who will go on to develop ALS ahead of the visible onset of symptoms and the variability of the disease course complicate efforts to study the early stages of the disease in humans, underscoring the need to use animal models to investigate pathogenic mechanisms of the disease.

The transgenic SOD1^G93A^ mouse, expressing ∼20 copies of human SOD1 with the G93A mutation, was developed shortly after SOD1 mutations were first linked to ALS (Gurney et al., 1994). It is currently still the model that best recapitulates several core clinical and neuropathological features of the disease. These mice invariably show progressive hind limb tremor and weakness, locomotor deficits, and paralysis followed by premature death (Bendotti and Carrì, 2004). In these models, extensive motor neuron death in the ventral horn is observed, along with the loss of myelinated axons in the ventral motor roots (Leitner et al., 2009). The mice present with progressive weakness in the hind limb leading to paralysis and death, almost perfectly replicating the disease process in patients (Gurney et al., 1994; Ripps et al., 1995; Wong et al., 1995). These models have been crucial in our understanding of the underlying pathophysiology of ALS (Bento-Abreu et al., 2010), identifying mechanisms linked to motor neuron death such as mitochondrial dysfunction, oxidative stress, protein aggregation, neuroinflammation, and axonal transport defects. Several preclinical trials have been performed in this mouse model, with some interventions successfully delaying the disease course (Carrì et al., 2006; Turner and Talbot, 2008; Mancuso and Navarro, 2015). Unfortunately, these have not led to successful clinical trials in ALS patients (Benatar, 2007; Mitsumoto et al., 2014).

The unpredictable severity of the disease in patients is a key factor in the many failed clinical trials in ALS. This has made it difficult to decipher pathogenesis and to develop effective therapeutic strategies. There is growing evidence indicating that disease severity in humans may be influenced by their genetic background, as seen in mutant SOD1 mice altering the cellular response to mutant SOD1 in a manner that either ameliorates or exacerbates the disease phenotype (Heiman-Patterson et al., 2011). Discovering the molecular mechanisms underlying the variability in the ALS progression may improve our understanding of modifiers of disease development.

In this study, we examined the effects of SOD1^G93A^ on the C57BL/6JOlaHsd (C57) and the 129SvHsd (129S) murine genetic backgrounds, i.e., in the C57-G93A and 129S-G93A strains, two ALS models with major clinical differences as previously described (Pizzasegola et al., 2009). These two transgenic strains have the same copy number of the human mutant SOD1 transgene, contain the same mutation, and express the same amount of mutant SOD1 protein in the spinal cord. However, the transgenic 129S-G93A mice exhibit a much faster rate of disease progression, with mean survival of 129 ± 5 days, compared to the transgenic C57-G93A mice, which have a mean survival of 180 ± 16 days (Pizzasegola et al., 2009). Transcriptome analysis of laser capture microdissected motor neurons from the spinal cord of each line has revealed a marked down regulation at disease onset of genes involved in mitochondrial function, protein degradation, and axonal transport in the fast-progressing transgenic 129S mice (Nardo et al., 2013). The slow-progressing transgenic C57-G93A mice, on the other hand, exhibited an upregulation of genes involved in the regulation of the inflammatory and immune response, supporting a role for genetic modifiers of the disease in determining the severity of disease progression.

Metabolic alterations have been identified as potential factors affecting the variability of the disease course in both ALS patients (Dupuis et al., 2011) and in the fast- and slow-progressing ALS mouse models (Pfohl et al., 2015; Nardo et al., 2016). To verify this hypothesis and identify potential pathways associated with disease progression in SOD1^G93A^ mice, we used Gas Chromatography-Mass Spectrometry (GC-MS) metabolomics to study metabolic changes in the spinal cord, the key affected tissue in ALS. We studied the slow- and the fast-progressing SOD1^G93A^ ALS mouse models, analyzing samples at three time points: before the appearance of visible symptoms (presymptomatic), at disease onset and at late stage of the disease. We aimed to examine how the metabolic response in the spinal cord evolves over the natural history of the disease, as well as to identify variations in metabolic responses to the mutant SOD1^G93A^ that may direct the two transgenic strains to lead different disease courses.

## Results

### Principal Component Trajectories of the Thoracic and Lumbar Spinal Cords Metabolomes in the Slow- and Fast-Progressing SOD1^G93A^ Mice

To investigate the dynamic variation in systematic metabolic responses to the mutant SOD1^G93A^-mediated disease process in the slow- and fast-progressing transgenic mouse strains, the metabolome was measured in the primary affected tissue in ALS, the spinal cord. We examined two spinal cord segments: the lumbar segment (which controls the hindlimbs and is affected earlier in disease) and the thoracic segment (which controls the axial muscles and is affected later in disease progression).

We examined multivariate patterns of metabolite variation and their relationship to disease progression in the spinal cord tissue metabolome by performing a PCA trajectory analysis. For each segment, we performed a principal component analysis (PCA) on the tissue metabolomes from all time points using log_10_-transformed, mean-centered and UV-scaled data. The principal component analysis describes the main patterns of variation in the metabolome data by calculating a series of principal components (PCs, linear combinations of the original descriptors – in this case the metabolite levels measured) where each PC is orthogonal to each other, allowing systematic variation in the highly multivariate metabolomic data to be summarized in PCs that describe related changes (Wold et al., 1987). The scores of the PCs were then examined to identify the presence of any intrinsic class-related patterns in the data. To visualize the trajectory of the multivariate patterns represented by the PCs over the stages of disease progression studied here, the scores of the principal components examined were averaged for each group of mice at each time point and plotted.

We looked at metabolic trajectories in the original principal components space (examining the scores as calculated in the principal components analysis), to allow for the examination of contributions of genetic background and SOD1 genotype differences to disease-related patterns in the temporal metabolic response. We also compared trajectory geometries between the different mouse genetic backgrounds and SOD1 genotypes by looking at aligned trajectories, where trajectories for all groups are shown to originate from the same starting point (by centering the mean scores of the pre-symptomatic time-points of each group to the origin). Examining the geometry of metabolic trajectories allows us to consider how similar the metabolic responses are between conditions, independent of inherent differences in the initial metabolic state (Keun et al., 2004).

The scale of trajectories were not adjusted for geometric comparisons, as no scalar enlargement or shrinkage of trajectories was observed between mouse backgrounds. This suggests that the overall magnitude of metabolic responses from mutant SOD1^G93A^ and over time are comparable between backgrounds, and that the difference lies more in the directionality of the metabolic response.

An analysis of variance of the linear model relating principal component analysis scores to the three experimental factors SOD1 genotype, background, and disease stage as well as their interactions was performed to determine the percentage of variance explained by each factor in the scores of each principal component. This indicated that variation in the first principal component was dominated by the linear model residuals, indicating that variation in PC1 is not being driven or strongly influenced by our experimental factors. Only small contributions from any of the other experimental factors were seen in PC1 (Supplementary Figure S1). As such, we focused the search for trends in metabolic trajectories in the subsequent principal components.

**FIGURE 1:**
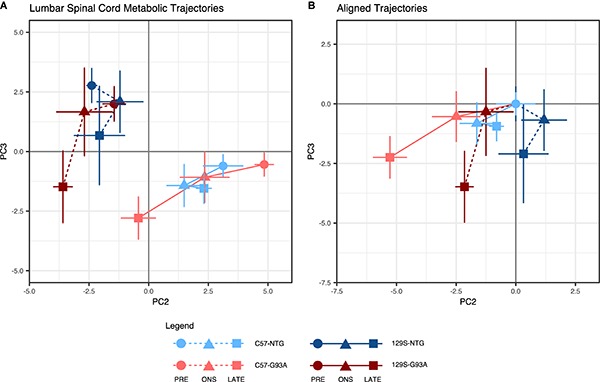
Lumbar spinal cord metabolic trajectories. Figure shows **(A)** metabolic trajectories from a principal components analysis of the lumbar spinal cord metabolome, and **(B)** aligned trajectories, where metabolic trajectories for each mouse background-SOD1 genotype combination are centered to their respective presymptomatic time points. Lumbar spinal cord metabolomes from non-transgenic (NTG) and transgenic human mutant SOD1^G93A^-expressing mice (G93A) in either the slower-progressing C57 background or the faster-progressing 129S background were measured at the presymptomatic stage (PRE), at disease onset (ONS), and at late stage (LATE). We examined the second (PC2) and third (PC3) principal components, which capture variation in the experimental factors being studied (percent contribution to variation of the experimental factors to each principal component are shown in Supplementary Figure S1A). The principal component scores for each group are shown as mean ± s.e.m. (*n* = 5 mice for each group per time point) for each time point.

In the lumbar spinal cord, there were pronounced differences in the metabolic profile between the two genetic backgrounds at all time points, with a clear separation between samples from the C57 and 129S mice along the PC2 axis (Figure 1A). The loadings show that the top metabolites contributing to PC2 scores are involved in alanine and aspartate metabolism, tyrosine and phenylalanine metabolism, and the malate-aspartate shuttle (Supplementary Figure S2A).

Trajectories for the SOD1^G93A^ mice of both backgrounds progressed toward the negative PC3 axis, ending at a comparable level at late stage. The NTG mice, on the other hand, traversed a much more limited distance over the three time points. This difference indicates the presence of a progressive metabolic response to expression of the mutant SOD1^G93A^ protein in the transgenic mice. The metabolites identified in the loadings to have the largest contributions to these effects in PC3 (Supplementary Figure S2B) include metabolites involved in central carbon metabolism, alanine, aspartate and glutamine metabolism, and branched chain amino acids.

When we examined the geometry of lumbar spinal cord metabolic trajectories, there were differing responses in the mice with C57 and 129S backgrounds (Figure 1B). In the C57 mice, the metabolic trajectories of the NTG and SOD1^G93A^ mice appear to traverse similar directions from the presymptomatic stage to onset, but diverge from each other leading into the late stage. In the 129S background, however, the NTG and SOD1^G93A^ mice exhibited opposing directions of response from the presymptomatic stage to onset.

The largest distance traversed on PC3 was from onset to late stage in both SOD1^G93A^ mice, with metabolic profiles at onset being comparable to their NTG counterparts. This suggests that there are no substantial metabolic changes in the lumbar spinal cord due to the effects of mutant SOD1 expression, and that metabolic responses in this tissue primarily occur in parallel to loss of motor function. Therefore, it is not clear whether they are cause or consequence of the disease, as they may reflect the metabolic state during large-scale motor neuron death. Motor neuron loss is comparable in C57-G93A and 129S-G93A mice (Marino et al., 2015).

In the thoracic spinal cord, we looked at trajectories in the PC2-PC4 space, where the differences between strains are not as pronounced in the NTG mice. A strong effect from mouse background was seen in PC3 (Supplementary Figure S1B), with a large positive-negative separation between backgrounds. Metabolic trajectory effects outside of those driven by mouse background were investigated by looking at PC4, which accounts for an almost equivalent proportion of variation in the data (11%) as PC3 (11.7%, Supplementary Figure S1B).

**FIGURE 2:**
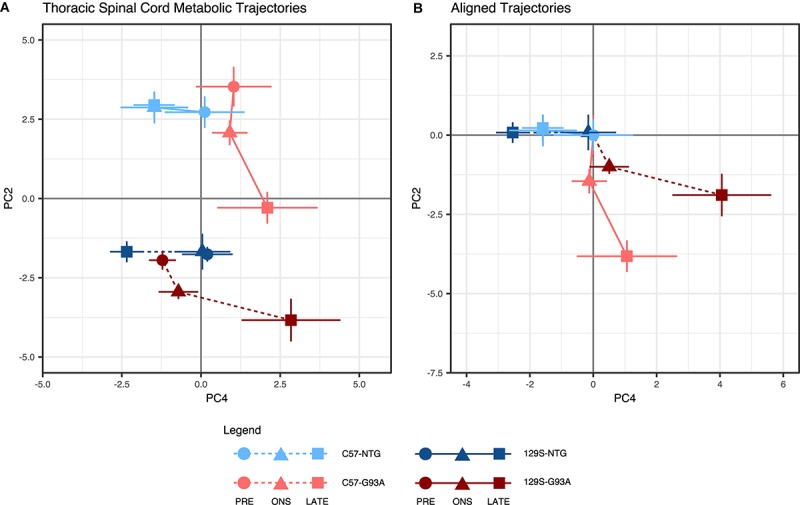
Thoracic spinal cord metabolic trajectories. Figure shows **(A)** metabolic trajectories from a principal components analysis of the thoracic spinal cord metabolome, and **(B)** aligned trajectories, where metabolic trajectories for each mouse background-SOD1 genotype combination are centered to their respective presymptomatic time points. Thoracic spinal cord metabolomes from non-transgenic (NTG) and transgenic human mutant SOD1^G93A^-expressing mice (G93A) in either the slower-progressing C57 background or the faster-progressing 129S background were measured at the presymptomatic stage (PRE), at disease onset (ONS), and at late stage (LATE). We examined the second (PC2) and fourth (PC4) principal components, which capture variation in the experimental factors being studied (percent contribution to variation of the experimental factors to each principal component are shown in Supplementary Figure S1B). The principal component scores for each group are shown as mean ± s.e.m. (*n* = 5 for each group per time point) for each time point.

Here, the metabolic trajectories for the NTG mice of both backgrounds were clustered around one quadrant of the PCA scores plot (Figure 2A). The SOD1^G93A^ mice had similar metabolic profiles to their NTG counterparts at the presymptomatic stage, but traversed away significantly from the NTG space at onset and late stage. Unlike in the lumbar spinal cord, the geometries of metabolic trajectories are much more similar in the thoracic spinal cord (Figure 2B). The metabolic trajectories of NTG mice are poorly defined and narrowly localized, while the SOD1^G93A^ mice have well-defined coincident trajectories away from the presymptomatic stage to onset, with divergence from onset to late stage.

The similarity of geometries suggests that the mode of response to SOD1^G93A^ in the thoracic spinal cord is comparable between the two mouse backgrounds. However, both the metabolic profiles and trajectories for the fast progressing 129S-G93A mice are shifted in the same direction as the disease-associated metabolic response. This suggests that in the thoracic spinal cord, the metabolic response to mutant SOD1 is the same, but that the initial metabolic state of the 129S-G93A mice are closer to that of the late disease state. Unlike the lumbar spinal cord, we observe a larger response in the thoracic spinal cord metabolic trajectories of both SOD1^G93A^ mice from the presymptomatic stage to onset, suggesting the presence of early metabolic responses (largely from PC2) that are mobilized in the tissue ahead of the presentation of visible symptoms. From the loadings, the main metabolites contributing to effects in PC2 (Supplementary Figure S3B), were amino acid metabolites and metabolites involved in ascorbate metabolism and carnitine synthesis.

Metabolic profile separation in the PC4 axis appears to be driven largely by SOD1 genotype, with NTG mice clustering toward the negative PC4 axis and mutant SOD1^G93A^ mice clustered toward the positive PC4 axis (Figure 2A). PC4 loadings indicate that the top contributors to variation in this component are neurotransmitters and neurotransmitter metabolites (Supplementary Figure S3A). We observe a distinctive disease response for the fast-progressing 129S-G93A mice compared to C57-G93A in terms of effects in this set of metabolites based on scores in PC4.

### Effects on Individual Metabolites in the Lumbar Spinal Cord

To evaluate effects on individual metabolites, we used a linear model incorporating effects from the three experimental factors: (1) SOD1 genotype, (2) mouse background, and (3) disease stage, as well as their two-way and three-way interaction effects (see section Materials and Methods).

**FIGURE 3:**
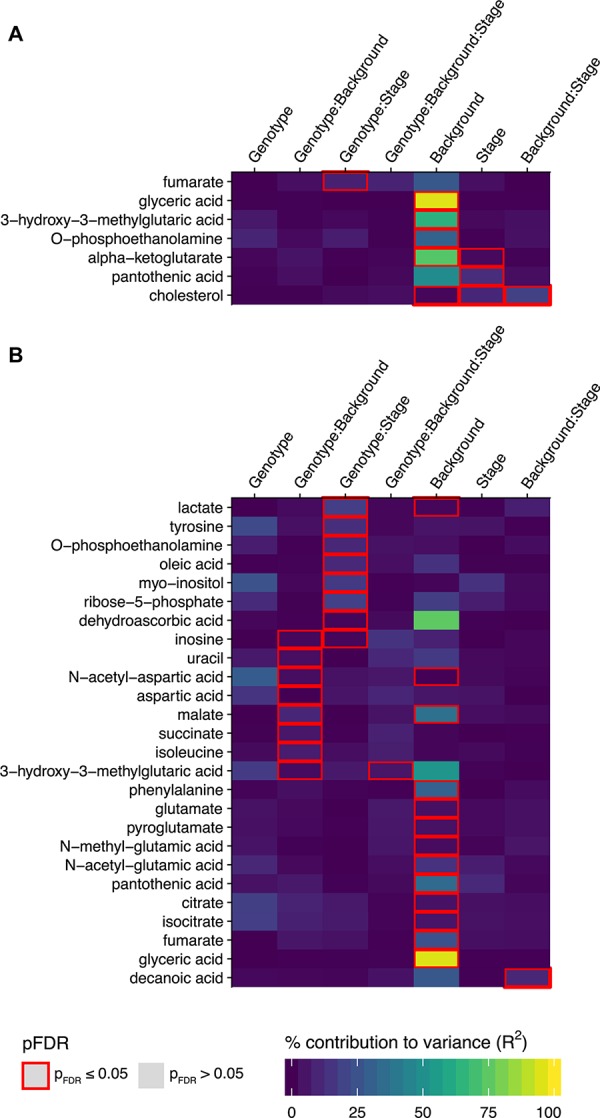
Metabolites with significant associations to SOD1 genotype, mouse background, disease stage, or their two-way and three way interaction effects in the linear model analysis. The percent contribution to variance (R^2^) for metabolites with significant associations to any of the three experimental factors SOD1 genotype, mouse background, and disease stage, or their two-way and three-way interaction effects in the linear model analysis (where p_FDR_ ≤ 0.05) are shown in heatmaps for **(A)** the lumbar spinal cord and **(B)** the thoracic spinal cord. Effects where *p* ≤ 0.05 after FDR multiple testing correction are outlined in red (*n* = 5 mice per group for each timepoint).

The largest percent contributions to variance in the metabolites significantly affected in the lumbar spinal cord were effects from mouse background, consistent with the clear clustering by mouse background observed in the metabolic trajectories (Figure 3A). There were significant effects from mouse background in 6 metabolites: glyceric acid, 3-hydroxy- 3-methylglutaric acid, O-phosphoethanolamine, alpha- ketoglutarate, pantothenic acid, and cholesterol (Figure 3A). Significant effects for disease stage were also observed for alpha-ketoglutarate, pantothenic acid, and cholesterol, as well as a significant effect for the interaction between background and disease stage for cholesterol. The only significant effect relating to SOD1 genotype in the lumbar spinal cord was in the interaction between SOD1 genotype and disease stage for fumarate.

The significantly affected metabolites in the lumbar spinal cord are involved in energy and lipid metabolism, with acetyl-CoA as a common node. Pantothenic acid is a key precursor in the synthesis of coenzyme A (CoA). Mitochondrial acetyl-CoA feeds carbon inputs from glycolysis into the TCA cycle, where we find significant effects in alpha-ketoglutarate and fumarate. Cytosolic acetyl-CoA is the ultimate precursor of fatty acids. It is therefore important in lipid metabolism, and there were significant effects in several intermediates: glyceric acid, a metabolite of the glycerol backbone of lipids, O-phosphoethanolamine, which forms the headgroup of PE-lipids, and cholesterol. 3-hydroxy-3-methylglutaric acid is produced from the degradation of leucine to 3-hydroxy-3-methylglutaryl-CoA (HMG-CoA), which can be cleaved into acetoacetate and acetyl-CoA. HMG-CoA is also involved in the synthesis of steroids such as cholesterol, due to its role as precursor in mevalonate synthesis.

**FIGURE 4:**
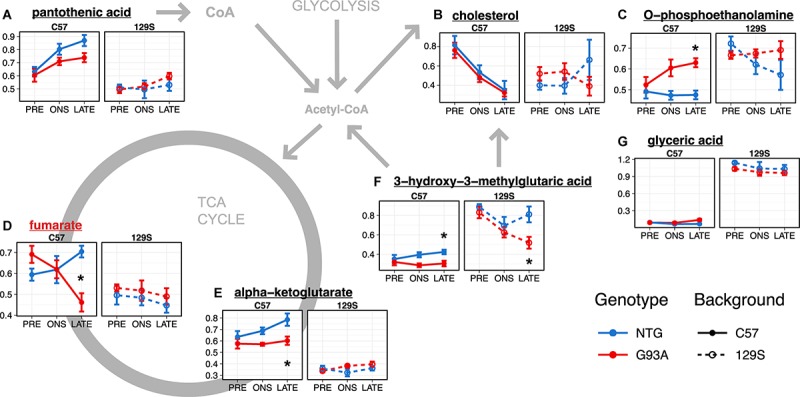
Metabolites with significant effects relating to mouse background or SOD1 genotype in the lumbar spinal cord **(A–G)** are involved in energy metabolism and lipid metabolism. Levels of metabolites with significant effects in the linear model analysis (as indicated in Figure 3A and Supplementary File S1) are shown as mean ± s.e.m. (*n* = 5 mice for each group per timepoint) of relative concentration (a.u.). Metabolites with significant effects relating to SOD1 genotype are highlighted with red names, while metabolites with background-related effects are listed in black. Asterisks denote comparisons between NTG and G93A mice where *p* < 0.05 and FDR < 20% from a *t*-test with Benjamini-Hochberg multiple testing correction (with full statistical results for the two-group comparisons in Supplementary File S2).

There were lower lumbar spinal cord levels of pantothenic acid (Figure 4A, *p* = 3.22 × 10^–9^, FDR < 5%, *t*-test comparing levels in C57 with 129S mice), alpha-ketoglutarate (Figure 4E, *p* = 5.11 × 10^–15^, FDR < 5%) and fumarate (Figure 4D, *p* = 3.09 × 10^–5^, FDR < 5%) in the 129S mice compared to the C57 mice. This may indicate less efficient TCA cycle activity and reduced CoA biosynthesis in mice with the 129S background. Levels of 3-hydroxy-3-methylglutaric acid in the 129S strain were higher than in the C57 strain (Figure 4F, *p* = 9.23 × 10^–13^, FDR < 5%), suggesting increased degradation of leucine as an energy compensation mechanism. The levels of O-phosphoethanolamine (Figure 4C, *p* = 6.10 × 10^–6^, FDR < 5%) and glyceric acid (Figure 4G, *p* = 3.20 × 10^–27^, FDR < 5%) were also higher in the 129S strain compared to the C57 strain. There were significant effects of background, stage, and their interaction in levels of cholesterol from the linear model (Figure 3A and Supplementary File S1), with similar levels in C57-NTG and C57-G93A mice that decreased over time (Figure 4B), contrasting with lower cholesterol levels in the 129S strain from the presymptomatic stage. Cholesterol levels in the presymptomatic C57 mice were significantly lower than in presymptomatic 129S (*p* = 3.554 × 10^–4^).

Overall, the major metabolomic changes in the lumbar spinal cord affected energy and lipid metabolism. These were mainly attributable to the background difference between the C57 and 129S strain, with differences between the transgenic C57-G93A and 129S-G93A mice observable at the presymptomatic stage.

### Effects on Individual Metabolites in the Thoracic Spinal Cord

We observed a greater number of significant effects in the thoracic spinal cord. There were significant effects relating to mouse background in 13 metabolites: lactate, N-acetyl-aspartate, malate, phenylalanine, glutamate, pyroglutamate, N-methyl-glutamate, N-acetyl-glutamate, pantothenic acid, citrate, isocitrate, fumarate, and glyceric acid (Figure 3B). A significant interaction effect between mouse background and disease stage was also observed for decanoic acid. There were also significant effects for interactions with SOD1 genotype in 15 metabolites. There were significant interaction effects between SOD1 genotype and disease stage for lactate, tyrosine, o-phosphoethanolamine, oleic acid, myo-inositol, ribose-5-phosphate, dehydroascorbic acid, and inosine. Inosine was also found to have significant interaction effects between SOD1 genotype and mouse background, along with uracil, N-acetyl-aspartate, aspartate, malate, succinate, isoleucine and 3-hydroxy-3-methylglutaric acid. Finally, the three-way interaction effect between SOD1 genotype, mouse background, and disease stage was also significant for 3-hydroxy-3-methylglutaric acid.

**FIGURE 5:**
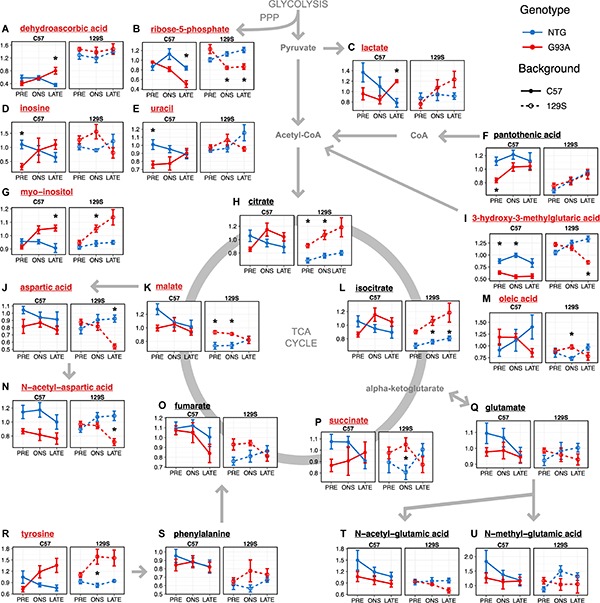
Metabolites involved in energy metabolism, anti-oxidant activity, and neurotransmitter amino acid metabolism **(A–U)** show significant effects in the thoracic spinal cord. Levels of metabolites with significant effects in the linear model analysis (as indicated in Figure 3B and Supplementary File S1) are shown as mean ± s.e.m. (*n* = 5 mice for each group per timepoint) of relative concentration (a.u.). Metabolites with significant effects relating to SOD1 genotype are highlighted with red names, while metabolites with background-related effects are listed in black. Asterisks denote comparisons between NTG and G93A mice where *p* < 0.05 and FDR < 20% from a *t*-test with Benjamini-Hochberg multiple testing correction (with full statistical results for the two-group comparisons in Supplementary File S2).

A number of these metabolites are involved in central carbon metabolism and energy production, like the glycolytic product lactate, the key CoA precursor pantothenic acid, 3-hydroxy-3-methylglutaric acid, and the TCA cycle intermediates citrate, isocitrate, succinate, fumarate and malate. Levels of these metabolites in the NTG mice tend to be higher under the C57 background compared to the 129S (*p* < 0.05 and FDR < 5% comparing levels in C57 mice to 129S for fumarate, malate, and pantothenic acid, Supplementary File S3) apart from 3-hydroxy-3-methylglutaric acid, where levels were generally higher under the 129S background (*p* = 8.11 × 10^–11^, FDR < 5%, Figure 5I). This may indicate lower activity of energy metabolism pathways and increased metabolism of leucine to acetyl-CoA that can enter the TCA cycle as a compensatory mechanism in the 129S-NTG mice. Levels of oleic acid (Figure 5M) were also markedly higher in the C57 mice compared to 129S (*p* = 0.0039, FDR < 5%).

When we go on to examine the effect of SOD1^G93A^ expression, we find that the two transgenic strains had different responses to mutant SOD1^G93A^ protein expression. Levels of lactate (Figure 5C) in SOD1^G93A^ mice were elevated compared to NTG at late stage under both backgrounds (*p* = 0.0056, FDR < 20%). We observed similar changes in thoracic spinal cord levels of 3-hydroxy-3-methylglutaric acid in the two transgenic strains to that in the lumbar spinal cord, with generally lower levels with little temporal variation in SOD1^G93A^ mice compared to NTG under the C57 background (*p* = 2.63 × 10^–7^, FDR < 5%, Figure 5I) contrasting with a steady decrease over time in the fast-progressing 129S-G93A mice. There were comparable levels of 3-hydroxy-3-methylglutaric acid in the 129S-G93A compared to 129S-NTG at the presymptomatic stage but with markedly lower levels at late stage (*p* = 3.06 × 10^–4^, FDR < 20%). Levels of citrate (Figure 5H) and isocitrate (Figure 5L) were lower in the C57-G93A mice compared to C57-NTG at presymptomatic stage and higher at onset and late stage, but these differences were not statistically significant (Figure 5). On the other hand, there were consistently higher levels of citrate (*p* = 1.13 × 10^–4^, FDR < 5%) and isocitrate (*p* = 1.37 × 10^–4^, FDR < 5%) in 129S-G93A mice compared to 129S-NTG, steadily increasing over time for both genotypes. Levels of succinate, fumarate, and malate tended to be lower in C57-G93A mice compared to C57-NTG, but tended to be higher in 129S-G93A compared to 129S-NTG (*p* = 0.0020 for malate).

The other large group of metabolites showing significant effects in the linear model were amino acids that act as neurotransmitters: aspartate, N-acetyl-aspartate, tyrosine, glutamate, and glutamate metabolites (Figure 3B). We observed significantly lower N-acetyl-aspartate levels in C57-G93A mice compared to C57-NTG (*p* = 3.32 × 10^–4^, FDR < 5%, Figure 5N). There was also a significant decrease in aspartate (*p* = 3.30 × 10^–4^, FDR < 20%, Figure 5J) and N-acetyl-aspartate (*p* = 0.0026, FDR < 20%, Figure 5N) levels of 129S-G93A mice compared to 129S-NTG at late stage, but no significant differences at presymptomatic stage and onset. N-acetyl-glutamate levels were also significantly lower in the 129S mice compared to C57 (*p* = 0.0038, FDR < 5%, Figure 5T). Levels of tyrosine tended to be higher in SOD1^G93A^ mice compared to NTG in both strains (*p* = 3.20 × 10^–4^, FDR < 5%, in the 129S mice, Figure 5R), with marked elevations in the SOD1^G93A^ mice from onset (significant in the 129S mice at onset, *p* = 0.0164, FDR < 20%). We also observed a progressive increase in thoracic spinal cord levels of myo-inositol in SOD1^G93A^ mice of both strains (Figure 5G), with significant differences between all C57-G93A and all C57-NTG mice (*p* = 0.0164, FDR < 20%), all 129S-G93A and all 129S-NTG mice (*p* = 0.0023, FDR < 5%) and as well between C57-NTG and C57-G93A mice at late stage (*p* = 0.0069, FDR < 20%) and between 129S-NTG and 129S-G93A mice at onset (*p* = 0.0111, FDR < 20%). Inositol has a diverse range of important function in neural tissues, as it is a key osmolyte in the CNS (Fisher et al., 2002) and a precursor for phosphoinositol lipids, which play a prominent role in signal transduction (Fisher et al., 1992) as well as facilitate cellular events such as regulating cell death and survival, membrane trafficking, and maintaining the actin cytoskeleton (Vanhaesebroeck et al., 2001).

The other significantly affected metabolites may indicate the presence of increased oxidative stress. Levels of ribose-5-phosphate (Figure 5B), an intermediate of the pentose phosphate pathway, were decreased in the SOD1^G93A^ mice compared to NTG in both mouse backgrounds at onset (*p* = 0.0013, FDR < 20% in the 129S mice) and late stage (*p* = 0.0053, FDR < 20% in C57 mice and *p* = 0.0023, FDR < 20% in 129S mice). Levels of inosine (Figure 5D) and uracil (Figure 5E) in NTG mice of both strains appear to be comparable, although levels were increased in 129S-G93A mice compared to C57-G93A mice (*p* = 6.07 × 10^–4^, FDR < 5% for uracil). Presymtomatic levels were also lower in C57-G93A mice compared to C57-NTG for inosine (*p* = 0.0038, FDR < 20%) and uracil (*p* = 0.0019, FDR < 20%), but no significant difference was seen in 129S mice. The presence of significant responses to SOD1^G93A^ expression in nucleotides may relate to metabolism of uric acid, which can function as an antioxidant (Hooper et al., 1998). There was also a broad increase in dehydroascorbic acid levels in 129S mice compared to C57 regardless of genotype and disease stage (*p* = 9.05 × 10^–19^, FDR < 5%, Figure 5A), indicating increased oxidation of the antioxidant ascorbic acid or broadly increased antioxidant demand under the 129S background.

Overall, the major metabolic changes in the thoracic spinal cord affected the related pathways of energy metabolism, neurotransmitter amino acid metabolism, and antioxidant homeostasis. Here, we observed effects attributable to the difference in genetic background the two mouse strains, as well as to interactions of SOD1^G93A^ protein expression with mouse genetic background or the stage of disease.

## Discussion

### Thoracic Spinal Cord Exhibits Large Metabolic Shifts Ahead of the Onset of Symptoms, While the Lumbar Spinal Cord Presents Few Metabolic Changes From Onset

We studied metabolomic changes in the spinal cord of two murine models of fALS with the same genetic trigger (mutant human SOD1^G93A^, expressed in the same amount), but different rates of disease progression. Metabolomic signatures in the spinal cord of these mice might relate to disease severity and help define currently unknown aspects of disease progression variability.

Results show that it is possible to distinguish spinal cord metabolomes in these models by disease stage. In the thoracic spinal cord, metabolomes of SOD1^G93A^ mice exhibited large deviations from their NTG counterparts between the presymptomatic stage and onset, regardless of mouse background. This indicates that the largest metabolic shifts in the thoracic spinal cord happen ahead of the onset of symptoms, and that a metabolic response to mutant SOD1 begins early in the life of the mouse. A greater understanding of early metabolic responses to mutant SOD1 may open possibilities for maintaining motor neurons viability. However, full characterization of these metabolic alterations poses a significant challenge as no clinical or biochemical parameters can conclusively identify early ALS before the presentation of motor symptoms.

It is possible that metabolic responses observed in the thoracic spinal cord may be limited to SOD1-fALS, or even to the subset with SOD1^G93A^, as disease development may vary with the SOD1 mutation present (Andersen et al., 1997). Other SOD1 ALS-related metabolic changes have previously been reported, including differences in cerebrospinal fluid metabolomes with SOD1-fALS patients (Wuolikainen et al., 2012), and neurometabolic changes in cervical spinal cords of asymptomatic mutant SOD1-positive individuals similar to those observed in patients with clinically apparent ALS (Carew et al., 2011). Studies of neonatal high copy number SOD1^G93A^ mice also demonstrate early and widespread abnormal neuronal activity and hyperexcitability (van Zundert et al., 2008).

The thoracic and lumbar spinal cord show similar histopathological changes in ALS patients (Schiffer et al., 1996; Wetts and Vaughn, 1996). A few studies have examined thoracic spinal cord involvement in the murine models. There were degenerative vacuolar changes in thoracic spinal cord motor neurons of the first SOD1^G93A^ mouse line without loss of those cells (Gurney et al., 1994; Chiu et al., 1995). There were no changes in proteasomal activities and no impairment of glucose use rates in the thoracic spinal cord at presymptomatic stage, while significant differences were already apparent in the lumbar tract (Kabashi et al., 2004; Browne et al., 2006). This suggests a slow involvement of the thoracic segment in the disease.

In the lumbar spinal cord, typically the principal region affected in ALS (Chen et al., 2010), the largest metabolic trajectory responses to SOD1^G93A^ expression occur from onset to late stage, with similar metabolic profiles for G93A and NTG mice at presymptomatic stage and onset. The onset of visible symptoms of ALS indicates that a critical mass of motor neurons has already been compromised (Sobue et al., 1983). Histopathological comparison of C57-G93A and 129S-G93A lumbar spinal cords mice showed statistically significant motor neuron loss at onset, and is comparable between the slow-progressing C57-G93A and fast-progressing 129S-G93A (45% vs. 48% motor neuron death), increasing up to 60 and 52% respectively at the late stage (Marino et al., 2015). As such, metabolic effects of SOD1^G93A^ in the lumbar spinal cord may reflect metabolic changes that occur with widespread motor neuron death sufficient to manifest as a motor loss phenotype. It should be emphasized that while the progressive loss of motor neurons from onset to late stage occurs in 2 weeks in fast-progressing mice, a similar loss occurs in 4 weeks in slow-progressing mice. This indicates that some compensatory mechanisms are activated in the motor neurons of slow-progressing mice to keep motor neurons alive for longer.

While we identified relatively few significantly affected metabolites in the lumbar spinal cord, previous work on lumbar motor neuron transcriptomes in the two models we analyze in this study showed a large number of gene expression changes (Nardo et al., 2013). However, those transcriptomes were of a single cell type. As numerous metabolic processes are compartmentalized in the CNS, the analytical approach in the present study examines the metabolome at organ region level which includes contributions from different constituent cell types. In this regard, previous work on the two mouse models studied here show that the environment surrounding the motor neurons is different, with higher microglia activation in the lumbar spinal cord of the faster-progressing 129S-G93A mice compared to C57-G93A, but similar levels of reactive astrocytosis (Marino et al., 2015; Nardo et al., 2016).

Results in the thoracic spinal cord present the possibility that prophylactic interventions to modify the early metabolic states of individuals susceptible or likely to develop ALS may delay the onset or progression of the disease. Preconditioning with latrepirdine, an adenosine 5′-monophosphate-activated protein kinase (AMPK) activator, delayed the onset of symptoms and extended lifespan in SOD1^G93A^ mice (Coughlan et al., 2015) but did not improve survival in SOD1^G93A^ mice when administered from symptom onset (Tesla et al., 2012). The impact of the presymptomatic metabolic state on disease course is further supported by delayed onset and improved survival with presymptomatic administration of other treatments such as withaferin A (Patel et al., 2015), guanabenz (Wang et al., 2014), and davunetide (Jouroukhin et al., 2012).

Our results introduce key considerations in the design of future spinal cord metabolism studies, as analysis of the thoracic spinal cord may be useful to characterize earlier biochemical abnormalities, while lumbar spinal cord tissue may provide key information on metabolic responses to significant motor neuron loss. Unlike in the lumbar spinal cord (Marino et al., 2015), we only found a significant reduction in the number of thoracic spinal cord motor neurons at the late stage, with no differences between the two mouse strains (Supplementary Figure S4A), supporting the later involvement of this region in disease progression in the SOD1^G93A^ mice. However, we observed significant astrogliosis and microgliosis activation in the absence of motor neuron loss in the thoracic segment of both SOD1^G93A^ mouse strains at onset (Supplementary Figures S4B,C). Considering that the microglia, but not astrocytes, are already activated at the presymptomatic stage in the lumbar spinal cord without inducing metabolomic changes, we suggest that astrocytosis or other mechanisms are involved in early metabolic abnormalities in the thoracic spinal segment. From this investigation of tissue metabolic changes, a more detailed dissection of these metabolic processes can be initiated, potentially using more advanced analytical techniques to better resolve cell type-specific contributions influencing disease severity.

### The Spinal Cord Metabolome Highlights Perturbations in Energy, Neurotransmitter, and Antioxidant Homeostasis Induced by Expression of Mutant SOD1^G93A^

Levels of intermediates and substrates of glycolysis and the TCA cycle fundamental for energy metabolism were altered in both the lumbar and thoracic spinal cord. This is consistent with the energy imbalance observed in mutant SOD1 mice, with signs of hypermetabolism observed weeks before the onset of symptoms (Dupuis et al., 2004). This has been attributed to abnormalities in muscle energy metabolism (Dupuis et al., 2011) and accompanied by expression of mitochondrial uncoupling proteins in skeletal muscle (Dupuis et al., 2003). However, early dysfunctions in energy metabolism have also been observed in CNS tissue, with abnormal mitochondrial morphology appearing as an early pathogenic feature in mutant SOD1 mice (Wong et al., 1995; Bendotti et al., 2001). Mitochondrial complex I activity was decreased in the SOD1^G93A^ mouse as early as 2 months (Jung et al., 2002), which leads to defects in oxidative phosphorylation and impaired ATP synthesis (Mattiazzi et al., 2002). SOD1^G93A^ mice have exhibited reduced glucose utilization in the brain prior to onset and in the spinal cord as the disease progressed (Browne et al., 2006), and impaired glycolysis in the lumbar spinal cord (Tefera and Borges, 2018). The drop in 3-hydroxy-3-methylglutaric acid levels (indicating increased branched-chain amino acid catabolism) we observe in the SOD1^G93A^ mice suggests decreased glycolysis, as the brain uses ketone as the primary energy source when energy requirements cannot be met by glucose (Kayer, 2006). We previously reported a marked decrease of mitochondrial transcripts and ATP production in the ventral portion of the lumbar spinal cord of 129S-G93A mice compared with C57-G93A at onset (Nardo et al., 2013). The unfavorable mitochondrial metabolic state observed in 129S-G93A mice compared with C57-G93A supports the idea that failing homeostatic regulation of these mechanisms may play a role in accelerating the disease (Irvin et al., 2015).

Furthermore, the metabolism of leucine (precursor of 3-hydroxy-3-methylglutaric acid) and other branched chain amino acids provides additional sources of acetyl-CoA. Breakdown of branched chain amino acids including leucine is increased in SOD1^G93A^ mice spinal cords (Tefera and Borges, 2018) and in SOD1^G93A^ mouse spinal neuron-astrocyte co-cultures (Valbuena et al., 2017).

Levels of amino acids involved in neurotransmission (glutamate and its derivatives, aspartic acid and N-acetyl-aspartic acid) were also perturbed in the thoracic spinal cord, consistent with previous reports both in ALS patients and SOD1^G93A^ fALS models. Decreased levels of aspartate and N-acetyl-aspartic acid have been observed using ^1^H MRS in patients (Foerster et al., 2013) as well as in SOD1^G93A^ mouse spinal cords and plasma (Niessen et al., 2007; Bame et al., 2014; Tefera and Borges, 2018). Glutamine-glutamate cycle homeostasis was altered in SOD1^G93A^ mouse astrocyte-spinal neuron co-cultures (Valbuena et al., 2017) and neuronal glutamate transfer to astrocytes was reduced in SOD1^G93A^ mouse spinal cords at disease mid-stage (Tefera and Borges, 2018). Glutamate is also essential for the synthesis of GSH, the main CNS antioxidant. Associated impairments in glutamate and GSH metabolism in a SOD1^G93A^ motor neuronal model were restored to control levels by supporting energy metabolism (D’Alessandro et al., 2011). Tyrosine levels, on the other hand, exhibited some of the largest changes and were elevated more quickly in the faster progressing 129S strain compared to the C57. The changes in the neurotransmitter pools will need to be examined in detail, particularly in the context of conflicting evidence on neuronal excitability, with studies demonstrating both hyperexcitability (Kuo et al., 2005; Pieri et al., 2009) and hypoexcitability (Delestree et al., 2014) in mutant SOD1 mouse neurons.

Other metabolic effects may reflect changes in antioxidant homeostasis with SOD1^G93A^ expression. Shifts in levels of nucleotides like inosine and uracil may indicate perturbation in metabolism of uric acid, a scavenger of peroxynitrite (formed when superoxide reacts with nitric oxide) and antioxidant (Hooper et al., 1998). Inosine supplementation increased bioenergetic output from glycolysis in astrocytes with the *C9Orf72* hexanucleotide repeat expansion and increased motor neuron survival in C9Orf72 ALS astrocyte-motor neuron co-cultures (Allen et al., 2019). Peroxynitrite amplifies oxidative damage in mutant SOD1 ALS (Drechsel et al., 2012), and nitrosative stress from SOD1 mutations contribute to protein aggregate formation, a neurotoxic mechanism (Basso et al., 2009). Increased uric acid levels were neuroprotective in a mouse model of multiple sclerosis, where they mitigated CNS tissue damage (Hooper et al., 1998).

The reduced levels of ribose-5-phosphate found with SOD1^G93A^ expression in the thoracic spinal cord are also consistent with increased levels of oxidative stress. This may indicate increased demand for reducing power in the form of NADPH, shifting ribulose-5-phosphate away from ribose-5-phosphate production toward the non-oxidative part of the pentose phosphate pathway, which produces NADPH. Maintenance of NADPH levels is fundamental to maintain GSH in the reduced form. Basal levels of dehydroascorbic acid were higher in the 129S-NTG, suggesting that oxidative stress levels may be higher in this strain and play a role in the faster rate of disease progression. This is supported by evidence that the 129S-NTG mice have higher O_2_ consumption and a higher basal metabolic rate than C57-NTG mice (Almind and Kahn, 2004).

### Metabolic Responses to Mutant SOD1^G93A^ in the SOD1^G93A^ Transgenic Mice Reflect Differences in Genetic Background Between the Two Strains

Some of the largest effects observed in the spinal cord tissue metabolomes relate to the different genetic background of the two mouse strains. This is the single largest discriminating factor in the lumbar spinal cord, and genetic background-related differences in thoracic spinal cord metabolic profiles are clear in all NTG mice and presymptomatic SOD1^G93A^ mice. These suggest that germline differences in metabolism may contribute to ALS progression differences between the two SOD1^G93A^ strains, as they may affect the capacity of the cell to address toxic impacts of mutant SOD1. This is consistent with the many signs of altered metabolic homeostasis seen in ALS patients (Ngo and Steyn, 2015).

The metabolic states of hyperlipidemia, high cholesterol levels, and type 2 diabetes positively correlated with survival in ALS (Dupuis et al., 2008; Seelen et al., 2014; Kioumourtzoglou et al., 2015) while weight loss associates with poor prognosis (Dupuis et al., 2011). In addition, a range of antecedent diseases have been associated with a delayed ALS onset age but a shorter disease duration (Hollinger et al., 2016). In this context, it is relevant that in wild type mice, the C57 genetic background renders the mice more prone to become obese, insulin resistant, and glucose intolerant and develop diabetes (Almind and Kahn, 2004; Mori et al., 2010). Interestingly, in SOD1^G93A^ mice, the C57 strain exhibits slower disease progression and lower weight loss compared to the 129S (Nardo et al., 2016). The significant associations of glycolysis and TCA cycle metabolites to genetic background we observe indicate impacts on energy and mitochondrial metabolism pathways. Taken together, these suggest that the slow-progressing C57-G93A mice may have a more favorable spinal cord bioenergetic profile than the 129S-G93A. Our study underlines a role for metabolism as a factor in determining disease onset age and survival length. However, further investigation is needed to determine the mechanisms by which these and other metabolic differences that arise due to differences in genetic background impact the severity of disease in ALS.

Thoracic spinal cord metabolomes of presymptomatic 129S-G93A mice appear to be more similar to metabolomes in symptomatic and advanced disease than to presymptomatic C57-G93A mice, indicating that the initial metabolic state under the 129S background may confer disadvantages in maintaining neuronal viability under ALS-related stressors. In instances where basal metabolic states produce similar effects to the response to an ALS mutant gene, a compounding effect may occur that accelerates disease progression. This would be consistent with ALS manifesting according to a liability threshold model, where disease develops when the burden of disease-causing factors crosses a critical threshold (Al-Chalabi and Hardiman, 2013). Analysis of ALS incidence rates indicate that the neurodegenerative process leading to ALS is a multistep process, with a large effect mutation accounting for a greater number of steps (Chió et al., 2018). The remaining steps needed to initiate disease are likely due to exogenous factors, involving an interplay between environmental factors and the genome of individual patients (Chió et al., 2018).

In the analysis of associations to tissue metabolism, we identify a number of metabolites with significant associations to background, stage, SOD1 genotype, or interactions between these factors but with a relatively low corresponding percentage contribution to variation in metabolite tissue levels (<10% for some metabolites). This is consistent with observations that contributions to variance in most metabolite levels studied are in the single digit percentages for household effects and significantly associated clinical covariates in families multiplex for coronary artery disease (Shah et al., 2009), for demographic variables (such as age, BMI, sex, ethnicity) in children from 6 european populations (Lau et al., 2018), for BMI in individuals from the TwinsUK and Health Nucleus studies (Cirulli et al., 2019), and for the combined contribution of clinical covariates in individuals from the Framingham study (Rhee et al., 2013). The fractions of variance associated with genetic traits in GWAS studies typically range from 1 to 12% (Gieger et al., 2008; Rhee et al., 2013) or less (Illig et al., 2010). SOD1 is not acting directly to produce metabolites observed in this study in cells, so it is not unreasonable that SOD1 genotype has a similar scale of contribution to variation in individual metabolites.

The significant background–related differences observed for several spinal cord tissue metabolites in the slow-and fast-progressing SOD1^G93A^ mice were seen in a well-defined ALS experimental model in a controlled setting where any environmental factors that may be involved in the disease are significantly reduced. This highlights the potential to identify cellular metabolic pathways that might change the disease course or serve as markers of disease severity in these murine models.

## Conclusion

This longitudinal study of the spinal cord metabolome in the slow-progressing C57-G93A and the fast-progressing 129S-G93A strain identifies the presence of metabolic responses to SOD1^G93A^ expression before the manifestation of visible symptoms (as seen in thoracic spinal cord tissue), as well as metabolic responses that are pronounced when there is an overt disease phenotype (as seen in the lumbar spinal cord tissue). We found substantial metabolic effects relating to differences in mouse genetic backgrounds, as well as effects that appear to relate mouse background to disease progression. The alterations to metabolite levels observed in the study may indicate changes to neurotransmitter synthesis and utilization, energy homeostasis, and the oxidative stress response. These results indicate a potential role for basal metabolic differences and their alteration of metabolic responses to mutant SOD1^G93A^ expression in influencing the disease severity.

Our investigation of the whole organ metabolome in the thoracic and lumbar segments of the spinal cord of SOD1^G93A^ strains with different disease progression has provided insights into the effect of early metabolic changes and background metabolic differences on variation in ALS disease progression. However, further experimental work is needed to delineate how each background may modify the cell metabolism of the different cell types in the spinal cord as well as their metabolic interplay in ALS. This underscores a need to characterize the impact of germline genetic variation on the cellular response to mutations in ALS genes in early life, to identify cellular processes that may delay the onset or progression of the disease.

## Materials and Methods

### Mouse Models

Female transgenic SOD1^G93A^ mice of the C57BL/6JOlaHsd (C57-G93A) or 129SvHsd (129S-G93A) genetic background and their corresponding non-transgenic (NTG) female littermates (C57-NTG and 129S-NTG respectively) were used in this study. These mouse lines were generated a few years ago from the original B6SJL-TgNSOD-1-SOD1^G93A^ -1Gur line expressing approximately 20 copies of human mutant SOD1 with a Gly93Ala substitution obtained from Jackson Laboratories and then maintained on a C57OlaHsd or 129SvHsd background (for more than 30 or 10 generations, respectively) at Harlan Italy S.R.L. We have focused our investigations on the C57-G93A and the 129S-G93A as we have observed marked differences in disease onset, progression and survival length between these models. The full characterization of the difference in disease onset, progression and survival length between the two SOD1^G93A^ strains with several functional outcome metrics have been detailed in previous publications (Pizzasegola et al., 2009). The age (in weeks, means ± S.D.) of the SOD1^G93A^ mice at the onset of symptoms, paralysis, and survival are shown in Supplementary Table S3.

Spinal cords were collected at the presymptomatic stage (at 8 weeks), at disease onset (19 weeks for the C57 strain, and 14 weeks for the 129S strain), and at late stage (21 weeks for the C57 strain, and 16 weeks for the 129S strain). Collection time points for each strain were determined on the basis of motor function analyses (paw grip strength test) and body weight. The progressive impairment of both fore- and hindlimb grip strength is one of the most widely used tests to measure disease progression (Ludolph et al., 2010).

The onset of symptoms was set as the age when the mice showed the first sign of paw grip strength impairment (reduced latency to fall from an inverted grid) and their body weight started to decline. The late stage was set as the point when the mice exhibited a decrease of ∼80% in their latency on the grip strength test and their body weight declined 20% from the initial value.

Mice were maintained at a temperature of 21 ± 1°C with a relative humidity of 55 ± 10% and a 12h light/dark cycle. Food (standard pellets) and water were supplied *ad libitum*. Procedures involving animals and their care were conducted according to the Mario Negri institutional guidelines, which are compliant with national (D.L. no. 116, G.U. suppl. 40, Feb.18, 1992, Circular No.8, G.U., 14 July 1994) and international policies (EEC Council Directive 86/609, OJ L 358, 1 Dec.12, 1987; NIH Guide for the Care and use of Laboratory Animals, U.S. National Research Council, 1996). All experiments and protocols were examined by the Institutional Ethical Committee and authorized by the Italian Ministry of Health. The mice were bred and maintained in a specific pathogen-free environment. Animals with substantial motor impairment had food on the cage bottom and water bottles with long drinking spouts.

### Tissue Preparation

Spinal cord tissues were obtained from C57-G93A and 129S-G93A mice and their corresponding age-matched NTG littermates (*n* = 5 for each group) at the presymptomatic, onset and late stage. Spinal cords were rapidly removed and placed in cold artificial CSF (127 mM NaCl, 1 mM KCl, 1.2 mM KH_2_PO_4_, 26 mM NaHCO_3_, 10 mM D-glucose, 2.4 mM CaCl_2_, 1.3 mM MgCl_2_) to limit metabolic processes prior to dissection. Tissues were then transferred onto a Petri dish cooled on ice, and the lumbar (L1-L6) and thoracic (T1-T13) segments dissected out and snap frozen in isopentane at −40°C. Tissues were stored at −80°C prior to metabolomic analysis.

### Metabolite Extraction

Approximately 25 mg of lumbar and thoracic spinal cord tissue was dissected on dry ice, weighed, and transferred to 2.0 mL screw cap tubes containing 0.1 mm glass beads. Metabolites were then extracted in 80% methanol using a Precellys24 tissue homogenizer operating at 6500 rpm in 2 cycles of 20 s. The resulting extract was dried down in a vacuum concentrator. Aqueous metabolites were then separated from the dried intracellular extract using a 2:1:3 chloroform:methanol:water extraction method. The aqueous portion of the extract was separated and lyophilized in silanized 1.5 mL glass vials prior to analysis.

### Gas Chromatography-Mass Spectrometry Metabolomic Analysis

Spinal cord metabolomes were analyzed in two separate batches, one for each spinal cord segment. Derivatization for GC-MS was carried out by methoximation followed by trimethylsilylation according to the protocol by Kind et al. (2009). Samples were analyzed on an Agilent 7890 gas chromatograph connected to an Agilent 5975 MSD using the FiehnLib settings (Kind et al., 2009) and retention time-locking to myristic acid-d27. GC-MS data were processed by deconvolution using AMDIS with the Fiehn library, followed by integration using GaVIN (Behrends et al., 2011) based on the quantitation ion for each metabolite as taken from the Fiehn library. Metabolite measurements were subjected to a smoothed spline normalization of repeat-injected pooled QC samples prior to further analysis.

### Statistical Analysis

Statistical analysis was carried out in R (3.4.3). Principal component analysis was performed on log_10_-transformed, mean-centered and unit variance (UV)-scaled data using the pcaMethods package. Measures of normality for each metabolite before and after log_10_-transformation are provided in Supplementary Table S1 for lumbar spinal cord metabolites and in Supplementary Table S2 for thoracic spinal cord metabolites.

The effects of experimental factors in our study were tested using a linear model relating tissue metabolite levels or principal components analysis scores to SOD1 genotype, mouse genetic background (strain), disease stage, and the two- and three-way interactions between these factors. The percentage of variance explained by each factor was determined using an ANOVA. The calculated *p*-values were adjusted for multiple comparisons to control the false discovery rate (FDR, Benjamini and Hochberg, 1995). The results of the linear model statistical analyses are provided in Supplementary File S1.

Two-group comparisons between NTG and G93A mice for each mouse background and disease stage were tested using a Student’s *t*-test, with *p*-values adjusted for multiple comparisons to control the FDR. The results of these statistical comparisons are provided in Supplementary File S2. Broader 2-group comparisons looking at larger subgroupings in the sample set (i.e., comparing all C57 with 129S mice, all C57-NTG mice with C57-G93A mice, etc.) were also tested using a Student’s *t*-test, with p-values adjusted for multiple comparisons to control the FDR. Comparisons where p_FDR_ ≤ 0.05 were considered significant. The results of these statistical comparisons are provided in Supplementary File S3. Comparisons where *p* ≤ 0.05 and FDR < 20% were considered significant; we have chosen to highlight FDR < 5% for most comparisons to focus on the most important changes. Summary statistics of metabolite measurements for each group are provided in Supplementary File S4.

### Immunohistochemistry and Motor Neuron Count

Mice at the onset of the symptoms, under deep anesthesia, were transcardially perfused with PBS followed by 4% PAF solution in PBS. The spinal cord was rapidly removed, post-fixed for 2 h and cryopreserved/dehydrated in 30% sucrose solution overnight before being frozen at −80°C. 10–12 sections of the thoracic spinal cord (segments T5–T8) of 4–5 mice per group were labeled with Neurotrace to detect the Nissl substance of neuronal cells (1:100 for 30 min, Life Technologies). Images were acquired with an Olympus Fluoview confocal microscope and neuron areas were analyzed with free software ImageJ. Only cells with an area ≥ 400 μm^2^ were considered for the quantitative analysis of motor neuron numbers. Data were expressed as mean number of motor neurons per section. Immunofluorescence was evaluated on five coronal thoracic spinal cord section (30 micron thickness) taken one every ten within the T5-T8 segment. After blocking the non-specific binding sites by incubation with a solution containing normal goat serum (NGS 10%) and Triton (0.1%) in PBS 0.01 M, the sections were incubated with the primary antibodies (overnight at 4°C), diluted in a solution containing NGS 1% and Triton 0.1% in PBS 0.01 M: mouse monoclonal anti-GFAP (1:2500, Merk Millipore), rabbit anti-IBA-1 (1:500, Wako). After three washes in PBS 0.01 M, the sections were incubated (1 h at room temperature) with appropriate secondary antibodies conjugated with a fluorophore (Alexa Fluor^®^ Dyes, Life Technologies), diluted (1:500) in a solution containing NGS (1%) in PBS 0.01 M. After 3 washes in PBS 0.01 M, the sections were mounted on glass slides and then covered with coverslips using FluorSaveTM (Calbiochem). Images were acquired with an Olympus Fluoview confocal microscope (20X magnification). The quantification of GFAP and IBA-1 intensity was carried out by determining the mean gray value of fluorescent signals in the gray matter of ventral horns, using the free software ImageJ.

## Data Availability Statement

The datasets generated for this study will be made available by the authors on request, without undue reservation, to any qualified researcher.

## Ethics Statement

Procedures involving animals and their care were conducted in accordance with the institutional guidelines at the Mario Negri Institute for Pharmacological Research, which are compliant with national (D.L. no. 116, G.U. suppl. 40, Feb.18, 1992, Circular No.8, G.U., 14 July 1994) and international (EEC Council Directive 86/609, OJ L 358, 1 Dec.12, 1987; NIH Guide for the Care and Use of Laboratory Animals, U.S. National Research Council, 1996) policies. All experiments and protocols were examined by the Institutional Ethical Committee and authorized by the Italian Ministry of Health.

## Author Contributions

GV carried out the metabolomics measurements and metabolomic data analysis. MT carried out the tissue collection for analysis. LC contributed to the design, analysis, and interpretation of the study. CB and HK conceived, designed, and coordinated the study. All authors discussed the results, wrote the manuscript, and read and approved the final manuscript.

## Conflict of Interest

The authors declare that the research was conducted in the absence of any commercial or financial relationships that could be construed as a potential conflict of interest.

## Supplementary Material

FIGURE S1 Percent contribution to variance of the experimental factors examined in the first 6 principal components of **(A)** lumbar spinal cord and **(B)** thoracic spinal cord metabolic profiles. Effects of the three experimental factors SOD1 genotype, mouse background, and disease stage, as well as their two-way and three-way interaction effects were examined using a linear model.

FIGURE S2 Lumbar spinal cord principal component analysis loadings for PC2 and PC3.

FIGURE S3 Thoracic spinal cord principal component analysis loadings for PC4 and PC2.

FIGURE S4 Neuropathological alterations in the thoracic segment of the spinal cord of two SOD1^*G93A*^ mouse strains and their respective non-transgenic mice. **(A)** Motor neuron count in thoracic spinal cord at different disease stages. Neurons were labeled with Neurotrace and motor neurons identified by the soma dimension (area ≥ 400 μm^2^). A slight but not significant decrease in the number of motor neurons was observed in both SOD1^*G93A*^ mouse strains compared to their respective non-transgenic (NTG) mice at the onset of symptoms (left) while this effect becomes more evident and significant in the late symptomatic stage (right). Quantification of **(B)** GFAP and **(C)** IBA-1 immunostaining in the ventral thoracic spinal cord of both SOD1^*G93A*^ mouse strains at the onset of the symptoms. In both strains, a marked increase of reactive astrocytosis (GFAP) and microglia (IBA-1) were observed compared to Ntg mice (two-way ANOVA, *n* = 4-5, ^∗^*p* < 0.05, ^∗∗^*p* < 0.01, ^∗∗∗^*p* < 0.001, and ^****^*p* < 0.0001).

TABLE S1 Shapiro-Wilk normality test results and skewness measures for the lumbar spinal cord data.

TABLE S2 Shapiro-Wilk normality test results and skewness measures for the thoracic spinal cord data.

TABLE S3 Age of mice at different points in the disease course. The onset of symptoms is defined as the time when mice show the first signs of limb muscle force deficit on grip strength (when they fall from the inverted grid before 90 seconds). Paralysis is defined as the time when mice are completely unable to stay on the inverted grid. Survival is defined when mice are not able to right themselves within 10 seconds when laid on their side.

FILE S1 Results of linear model statistical analysis.

FILE S2 Results of two-group comparisons (Student’s *t*-test) between NTG and G93A mice for each mouse background and disease stage.

FILE S3 Results of two-group comparisons (Student’s *t*-test) between larger subgroupings of the sample set.

FILE S4 Summary statistics of metabolite measurements for each group.
