# Supplementary material for: Spinal Cord Metabolic Signatures in Models of Fast- and Slow-Progressing SOD1G93A Amyotrophic Lateral Sclerosis
Source: Front Neurosci. 2019 Dec 10;13:1276. doi: 10.3389/fnins.2019.01276 (PMC6914819; doi:10.3389/fnins.2019.01276)
Supplement: FIGURE S1 — Percent contribution to variance of the experimental factors examined in the first 6 principal components of (A) lumbar spinal cord and (B) thoracic spinal cord metabolic profiles. Effects of the three experimental factors SOD1 genotype, mouse background, and disease stage, as well as their two-way and three-way interaction effects were examined using a linear model. [file Table_1.pdf]

Supplementary Material

1 Supplementary Figures

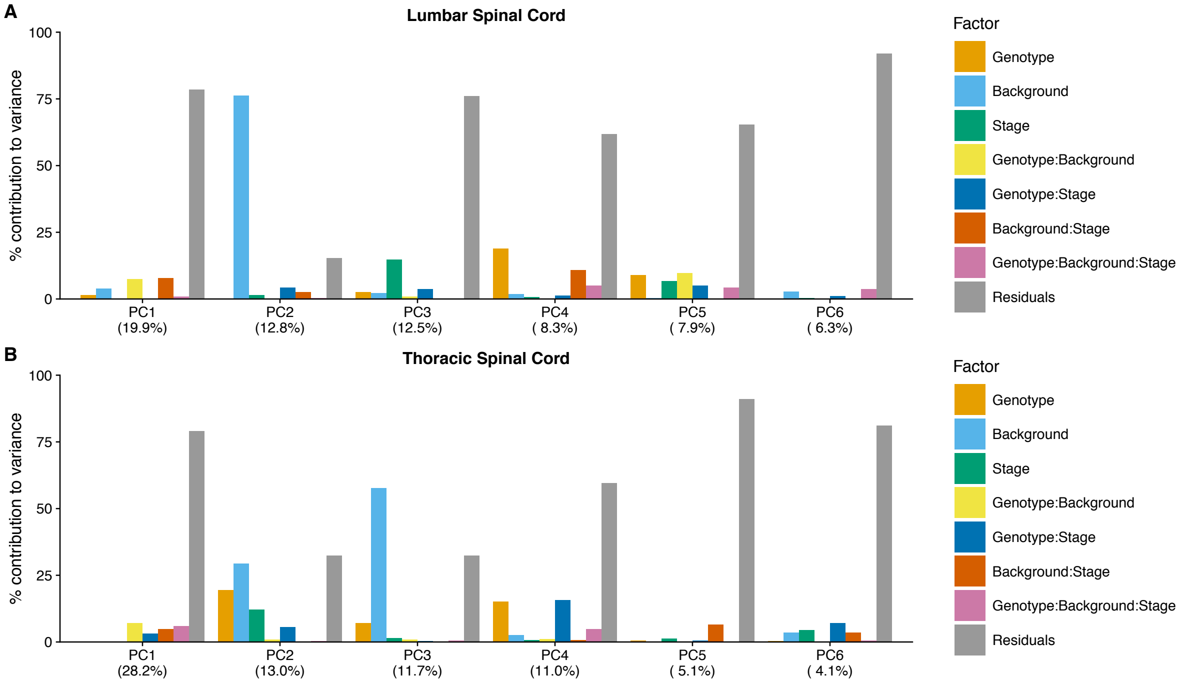

Supplementary Figure 1. Percent contribution to variance of the experimental factors examined in the first 6 principal components of (A) lumbar spinal cord and (B) thoracic spinal cord metabolic profiles. Effects of the three experimental factors SOD1 genotype, mouse background, and disease stage, as well as their two-way and three-way interaction effects were examined using a linear model.

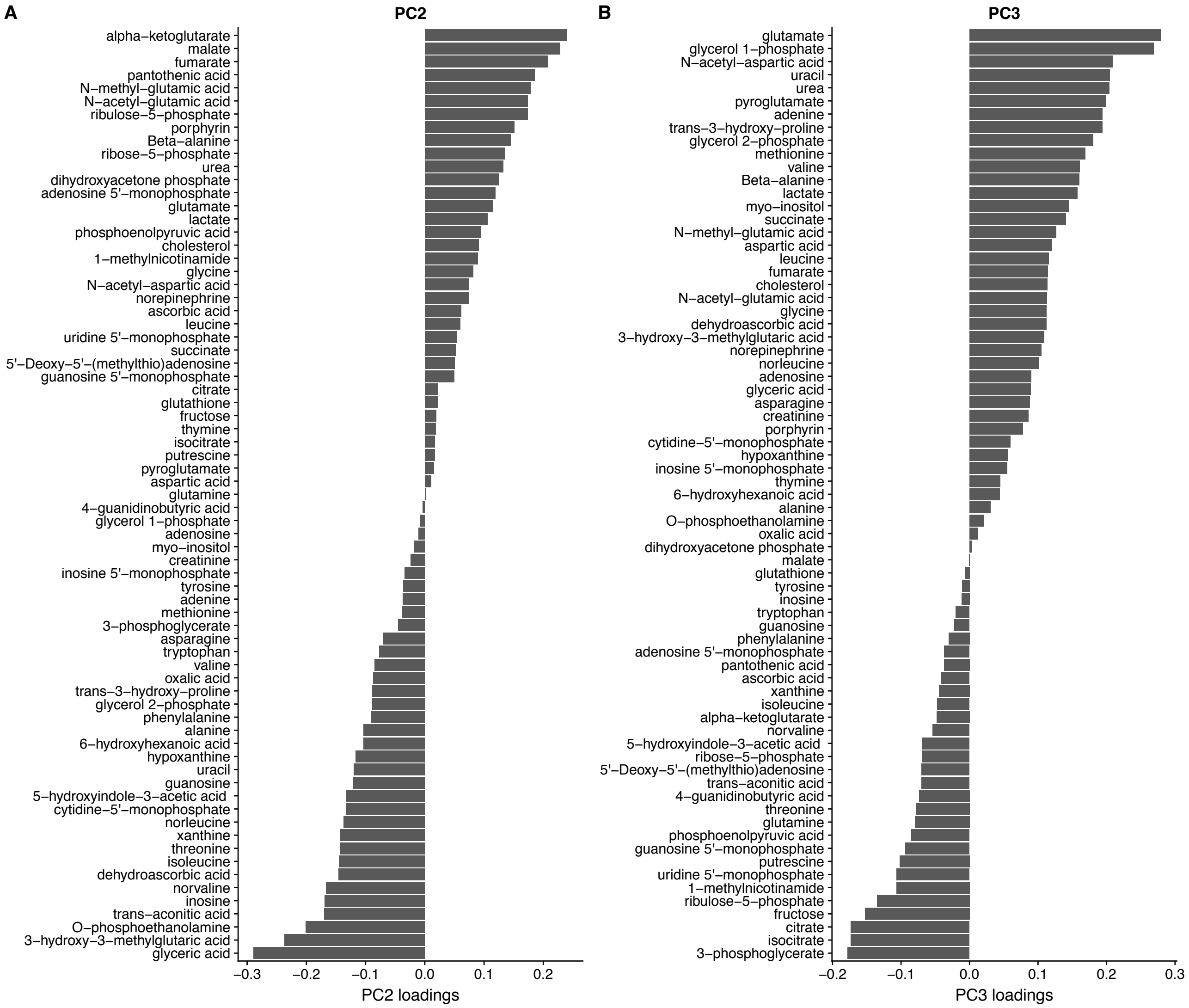

Supplementary Figure 2. Lumbar spinal cord principal component analysis loadings for PC2 and PC3.

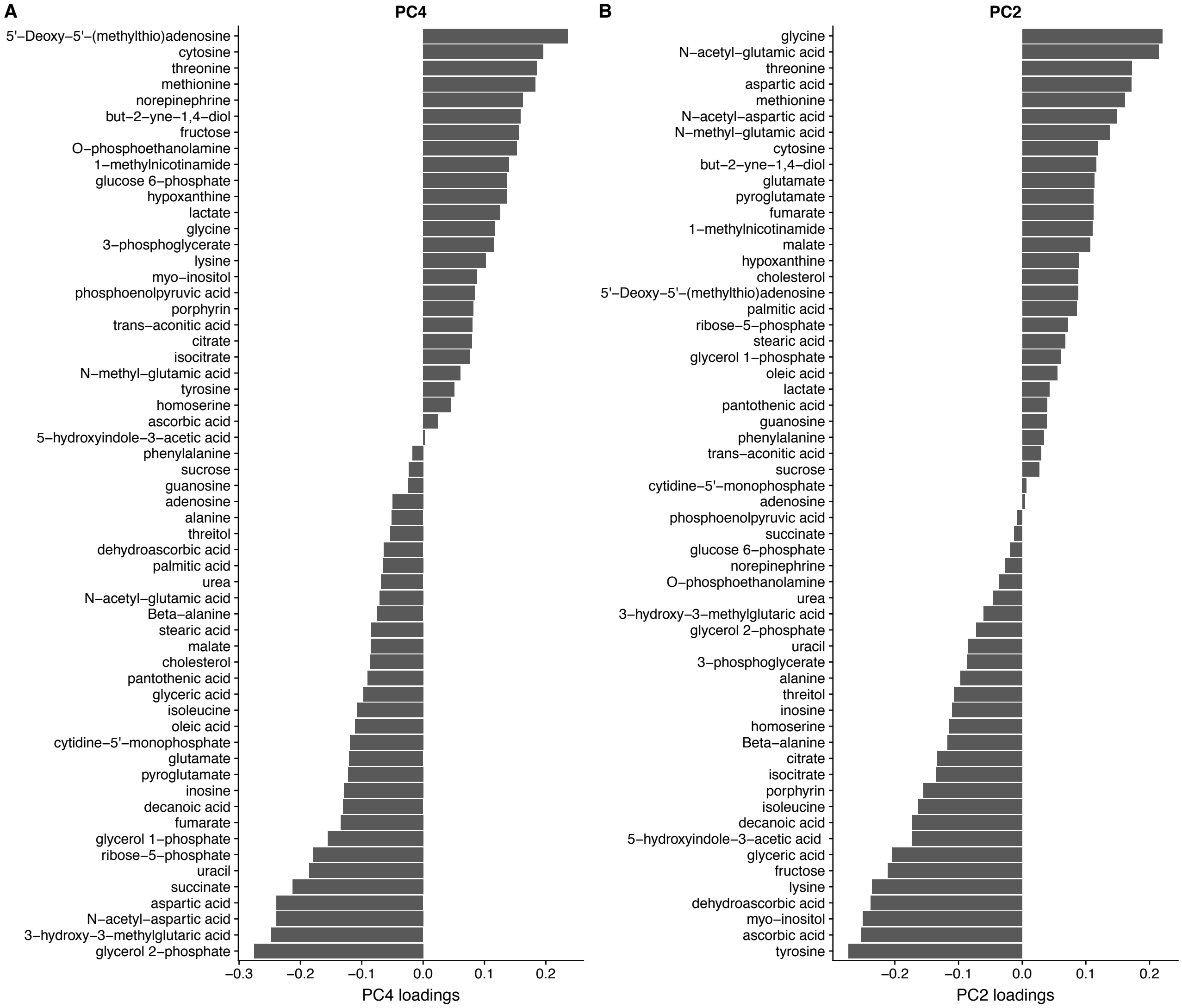

Supplementary Figure 3. Thoracic spinal cord principal component analysis loadings for PC4 and PC2.

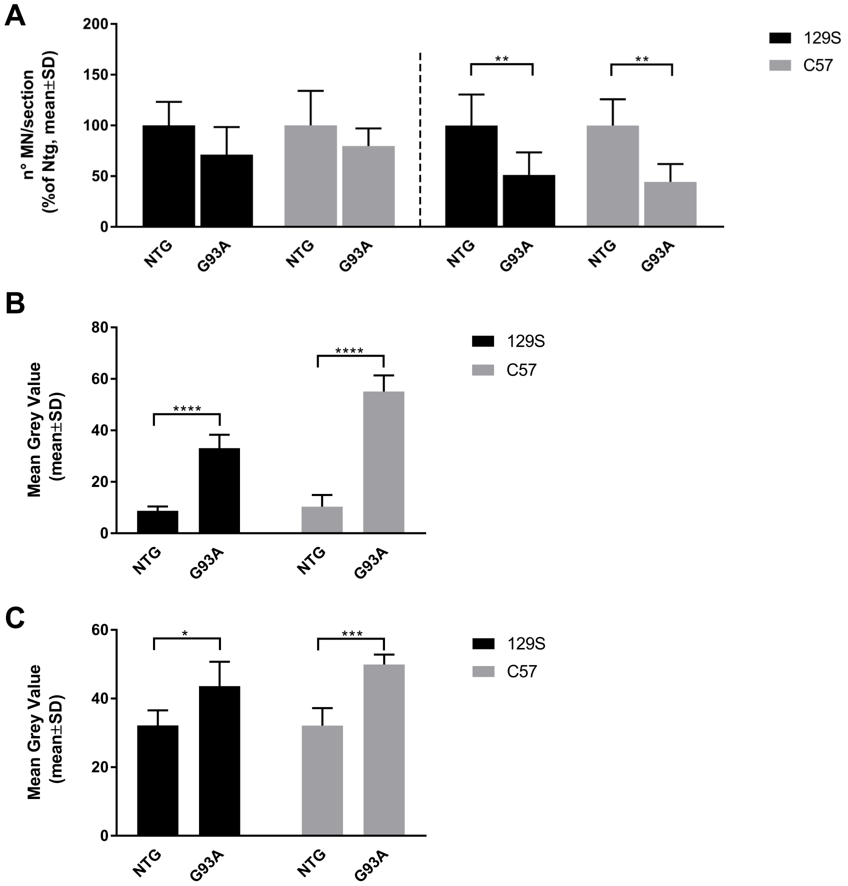

Supplementary Figure 4. Neuropathological alterations in the thoracic segment of the spinal cord of two SOD1^{G93A} mouse strains and their respective non-transgenic mice. (A) Motor neuron count in thoracic spinal cord at different disease stages. Neurons were labeled with Neurotrace and motor neurons identified by the soma dimension (area ≥ 400 μm^2). A slight but not significant decrease in the number of motor neurons was observed in both SOD1^{G93A} mouse strains compared to their respective non-transgenic (NTG) mice at the onset of symptoms (left) while this effect becomes more evident and significant in the late symptomatic stage (right). Quantification of (B) GFAP and (C) IBA-1 immunostaining in the ventral thoracic spinal cord of both SOD1^{G93A} mouse strains at the onset of the symptoms. In both strains, a marked increase of reactive astrocytosis (GFAP) and microglia (IBA-1) were observed compared to Ntg mice (two-way ANOVA, n=4-5, * $p < 0.05$, ** $p < 0.01$, *** $p < 0.001$, **** $p < 0.0001$).

2 Supplementary Tables

Supplementary Table 1. Shapiro-Wilk normality test results and skewness measures for the lumbar spinal cord data

metabolite	Untransformed data		log_{10}-transformed data	
	Shapiro-Wilk p-value	Skewness	Shapiro-Wilk p-value	Skewness
lactate	0.02040102	0.44774212	0.00355528	-0.6788974
norvaline	0.02464713	-0.4483184	4.00E-07	-1.5736805
oxalic acid	0.03182764	0.73002326	0.15238701	-0.3740703
norleucine	0.22865667	0.29792803	0.08599242	-0.3844879
isoleucine	0.07870787	-0.194213	0.00043436	-0.7691025
valine	0.01442641	0.87469199	0.5125091	0.43932992
urea	0.00209265	0.18477593	2.50E-05	-1.2983575
glycine	0.55609293	-0.3125919	0.03186714	-0.7058836
succinate	0.72650947	-0.2178467	0.06231515	-0.7016515
glyceric acid	3.97E-08	0.08127738	7.41E-08	-0.1194463
porphyrin	0.00022435	1.2741266	0.7901108	0.16837924
uracil	0.38417161	0.4016033	0.64881809	-0.2395524
fumarate	0.64582708	0.168718	0.26970727	-0.3495089
threonine	0.00069051	0.89336327	0.00413958	-0.4034829
thymine	0.06173438	0.14990475	0.00244254	-0.9002933
6-hydroxyhexanoic acid	0.15555482	0.44086269	0.20398432	-0.497403
Beta-alanine	2.63E-05	1.5661566	0.45093345	0.39933794
trans-3-hydroxy-proline	0.0003028	1.22474793	0.92550157	0.15237982
1-methylnicotinamide	1.71E-05	1.55691341	0.53396489	0.18708315
malate	0.22805966	0.10450474	0.10857986	-0.4573087
pyroglutamate	0.00075429	-1.1266884	1.63E-06	-1.9948463
4-guanidinobutyric acid	3.51E-07	1.92177285	0.60895133	-0.2123755
glutamine	0.63885483	-0.2750856	0.0049358	-1.0176923
phenylalanine	0.21478261	-0.4785612	0.00021901	-1.3340476
creatinine	0.00497969	0.31149342	0.00555907	-0.0821925
alpha-ketoglutarate	0.00256776	0.53439296	0.02108272	-0.0188888
phosphoenolpyruvic acid	6.32E-05	1.2353991	0.66915803	0.02396782
3-hydroxy-3-methylglutaric acid	0.00049511	0.48511189	0.01957794	0.00291231
N-acetyl-aspartic acid	0.01357195	-0.7379926	9.00E-05	-1.2114178
asparagine	0.01008885	0.39618716	0.07668982	-0.3252969
N-methyl-glutamic acid	0.51435831	0.06729656	0.00050828	-0.9631502
glycerol 2-phosphate	0.67180874	-0.2605035	0.04287739	-0.7051435
putrescine	7.98E-08	2.61618422	0.01309887	0.48321562
trans-aconitic acid	0.72658647	0.17391183	0.26853147	-0.4373365

glycerol 1-phosphate	0.01282367	-0.4965239	0.00023376	-1.0986612
O-phosphoethanolamine	0.02209108	-0.2583668	0.00224189	-0.5195303
N-acetyl-glutamic acid	0.53780941	-0.2453589	0.00473258	-0.9031522
citrate	0.00976504	0.22069631	0.00050769	-1.0082123
isocitrate	0.01233934	0.21062277	0.00054206	-1.0199078
adenine	0.02718839	-0.6319295	0.00065156	-1.1663856
fructose	7.77E-06	1.69792933	0.12990974	0.38651585
dehydroascorbic acid	0.28201975	0.45131139	0.31670763	-0.3552899
tyrosine	0.00036111	1.03889042	0.02135616	0.60295468
ascorbic acid	0.04477959	0.62498373	0.47374722	-0.3957052
pantothenic acid	0.56080716	0.33297749	0.75020579	-0.24484
xanthine	0.01102294	0.87466317	0.87894989	0.21124864
cytidine-5'-monophosphate	0.11455722	0.65833491	0.69385748	-0.165582
myo-inositol	0.00830092	-0.7775153	5.50E-06	-1.8797719
ribose-5-phosphate	0.01889108	0.85869809	0.91002644	-0.1198442
ribulose-5-phosphate	0.00451063	0.88954007	0.59350212	0.07388209
norepinephrine	0.54534728	-0.1697139	0.07993152	-0.5536569
glutathione	0.00346837	-0.4618695	2.16E-05	-0.9943321
tryptophan	2.99E-06	1.64830312	0.23277442	0.33238321
5-hydroxyindole-3-acetic acid	0.12365573	0.60737575	0.74893846	-0.0344799
inosine	0.0223292	0.65371995	0.73814891	-0.1032131
adenosine	0.00253102	1.04159204	0.97090648	0.02421294
guanosine	0.06175975	0.28783915	0.06950303	-0.4048177
5'-Deoxy-5'-(methylthio)adenosine	0.00358778	-0.6534773	2.72E-05	-1.2359627
uridine 5'-monophosphate	0.81084782	0.18233267	0.08355543	-0.6625947
inosine 5'-monophosphate	0.06964811	0.24637823	0.12931126	-0.2657276
adenosine 5'-monophosphate	0.5188134	0.05366184	0.1510513	-0.4701019
guanosine 5'-monophosphate	0.30649007	0.4150819	0.20468945	-0.4982112
cholesterol	0.0029408	0.92322202	0.85748265	-0.1047519
alanine	0.67331689	-0.106838	0.15436266	-0.5234367
leucine	1.35E-05	1.42286619	0.07287744	0.61958219
methionine	0.00292055	0.94984167	0.12459749	0.55635668
aspartic acid	0.05443855	0.06704621	0.00749326	-0.5719846
glutamate	0.43861664	-0.154033	0.0065909	-0.939417
dihydroxyacetone phosphate	0.02955529	0.67350542	0.52236933	-0.0830992
hypoxanthine	0.00505988	0.87834761	0.67246834	0.22446778
3-phosphoglycerate	0.65294438	0.29041497	0.54690206	-0.4257234

Supplementary Table 2. Shapiro-Wilk normality test results and skewness measures for the thoracic spinal cord data

metabolite	Untransformed data		log_{10}-transformed data	
	Shapiro-Wilk p-value	Skewness	Shapiro-Wilk p-value	Skewness
lactate	0.00103379	1.05339731	0.69415938	0.25179422
alanine	0.00735564	0.7498704	0.4241388	0.22380023
isoleucine	0.00902094	0.97468241	0.79362335	0.22874703
but-2-yne-1,4-diol	1.56E-06	1.13961873	0.00070159	0.42476312
urea	0.37492863	0.39270642	0.80549331	-0.1209646
threonine	1.99E-06	1.52165647	0.04236388	0.46762387
glycine	1.74E-05	1.68067649	0.68504592	0.24298625
succinate	0.81674464	0.01967431	0.33271641	-0.3921392
glyceric acid	1.13E-07	0.10065179	1.15E-07	-0.0529294
porphyrin	0.0225825	0.67975201	0.17895658	-0.0556902
uracil	0.01304499	0.64992778	0.07069526	-0.1991665
fumarate	0.96235452	0.18759787	0.90898562	-0.2733364
methionine	6.27E-12	3.12073146	8.58E-07	1.68519544
aspartic acid	0.95376795	0.02241364	0.25248719	-0.5241439
Beta-alanine	5.32E-08	1.96812002	0.05310032	0.65913551
homoserine	4.61E-16	4.49694867	1.32E-14	3.90106115
decanoic acid	0.07447819	-0.177188	0.02789525	-0.4022251
malate	0.06701654	0.63739183	0.8461334	0.13875674
1-methylnicotinamide	3.14E-09	2.376629	0.00393005	0.92504824
threitol	1.54E-05	-1.6660659	3.66E-12	-4.8243021
glutamate	0.0386482	0.68017333	0.28864605	0.2969733
pyroglutamate	0.1428269	0.50525941	0.48051248	0.17446407
cytosine	5.53E-08	2.04261499	6.00E-05	1.29368578
phenylalanine	0.12623879	0.52685874	0.82092098	-0.0121978
phosphoenolpyruvic acid	0.00151762	1.00933	0.59301599	0.34488983
3-hydroxy-3-methylglutaric acid	0.1784219	-0.0501328	0.01469032	-0.5217882
N-acetyl-aspartic.acid	0.29642219	0.33925689	0.43162884	-0.1655918
N-methyl-glutamic acid	0.00016299	1.4441075	0.52276049	-0.0833892
glycerol 2-phosphate	0.02528366	-0.0908842	0.01445822	-0.2710657
trans-aconitic acid	8.11E-08	1.76259298	0.00049528	0.99526882
glycerol 1-phosphate	0.0638909	0.62145339	0.46175349	-0.1399812
O-phosphoethanolamine	0.00073611	0.99772177	0.0291486	-0.1319156
N-acetyl-glutamic acid	9.93E-07	2.21121056	0.11275192	0.62598278
hypoxanthine	1.33E-12	3.12954232	0.00034524	1.08934277
3-phosphoglycerate	0.35351395	0.09395988	0.1245176	-0.4833503

citrate	0.02929924	0.65182437	0.70297506	0.20787004
isocitrate	0.03314647	0.6463238	0.72094896	0.20662518
dehydroascorbic acid	0.00222611	0.17407475	0.00134397	-0.4153103
fructose	8.42E-08	2.15137802	0.00114342	1.06642477
lysine	5.01E-10	2.99524522	0.01466412	0.84084986
tyrosine	2.24E-05	1.06077351	0.02207511	0.44155422
ascorbic acid	5.35E-10	1.93218434	0.5761648	0.04665431
pantothenic acid	0.14802669	0.47392104	0.76992519	0.063144
cytidine-5'-monophosphate	0.54582839	0.18660501	0.39166289	-0.2863956
palmitic acid	0.00039965	1.12812855	0.03730601	0.60340087
myo-inositol	0.0006933	1.09129241	0.01024614	0.84003518
ribose-5-phosphate	0.54810443	-0.1480995	0.00014226	-1.437473
norepinephrine	2.99E-06	1.5987109	0.01262846	0.43267124
5-hydroxyindole-3-acetic acid	0.01695962	0.75991055	0.72221248	0.10595959
oleic acid	3.18E-05	1.50818689	0.24663216	0.3142163
stearic acid	1.35E-07	2.44299905	0.00032749	1.32640923
glucose 6-phosphate	1.74E-08	2.06391493	0.92507777	0.09146722
inosine	0.03763147	0.7585465	1.39E-06	-1.8377256
adenosine	2.83E-06	1.78126616	0.85277155	0.01798009
sucrose	1.60E-13	4.64824812	1.40E-06	1.77803571
5'-Deoxy-5'-(methylthio)adenosine	2.05E-06	1.74925288	0.05461372	0.6162538
guanosine	6.94E-05	1.39443975	0.00041203	-0.9303759
cholesterol	7.45E-13	4.7433487	0.07131249	0.68735738

Supplementary Table 3. Age of mice at different points in the disease course. The onset of symptoms is defined as the time when mice show the first signs of limb muscle force deficit on grip strength (when they fall from the inverted grid before 90 seconds). Paralysis is defined as the time when mice are completely unable to stay on the inverted grid. Survival is defined when mice are not able to right themselves within 10 seconds when laid on their side.

Stage of disease	Weeks of age	
	129S-G93A	C57-G93A
Onset	14.3 ± 1.1	17.4 ± 1.0
Paralysis	16.1 ± 0.7	22.7 ± 1.5
Survival	17.8 ± 0.8	25.8 ± 1.6
